# Alogliptin Reduces Oxidative Stress in Cardiomyocytes and Ameliorates Diabetic Cardiomyopathy via the AURKB/NLGN2 Signaling

**DOI:** 10.1002/kjm2.70149

**Published:** 2025-12-22

**Authors:** Li‐Jing Jiao, Jing Zhou, Jing Wang, Si‐Nan Zhao, Zhan‐Sheng Zhao, Qian Wang, Ya‐Nan Xie, Wei Jiao

**Affiliations:** ^1^ Department of Endocrinology The Second Hospital of Hebei Medical University Shijiazhuang Hebei People's Republic of China; ^2^ Department of Cardiology The Second Hospital of Hebei Medical University Shijiazhuang Hebei People's Republic of China

**Keywords:** Alogliptin, AURKB, diabetic cardiomyopathy, NLGN2, oxidative stress

## Abstract

Diabetic cardiomyopathy (DCM) is a common complication of diabetes mellitus. This study investigated the effects of alogliptin on DCM and its underlying mechanisms. A DCM model was constructed and treated with alogliptin. Downstream targets of alogliptin were screened using bioinformatics analysis. An in vitro DCM model was constructed using mouse cardiomyocytes with a high concentration of glucose. Echocardiography was performed to measure the heart function parameters. Myocardial damage, collagenous fibrosis, and apoptosis of cardiomyocytes in mouse heart tissues were assessed using cardiac histological staining. AURKB and NLGN2 levels, ROS levels, MDA levels, SOD activity, and cardiomyocyte viability were determined. Alogliptin ameliorated DCM in mice. Bioinformatics analysis revealed that the target of alogliptin was AURKB, and the downstream target of AURKB was NLGN2. AURKB and NLGN2 levels were reduced in the heart tissues of streptozotocin‐induced mice. Combined knockdown of AURKB and NLGN2 inhibited the therapeutic effect of alogliptin in DCM mice. Alogliptin attenuated oxidative stress, increased viability, and decreased apoptosis in cardiomyocytes treated with high glucose, which were reversed by combined knockdown of AURKB and NLGN2. Overall, alogliptin ameliorated oxidative stress in cardiomyocytes and DCM in mice by promoting AURKB expression to transcriptionally activate NLGN2.

## 1 Introduction

As a result of dysregulated glucose and lipid metabolism associated with diabetes mellitus, diabetic cardiomyopathy (DCM) is a pathophysiological condition of cardiac structure and function changes in patients with diabetes that cannot be explained by coronary artery disease, hypertension, and other types of heart diseases [1, 2]. Hypoglycemic drugs, including metformin, sodium glucose cotransporter 2 inhibitors, dipeptidyl peptidase‐4 (DPP‐4) inhibitors, and thiazolidinediones, are effective in alleviating DCM [3]. Oxidative stress is considered a key component of its pathogenesis; thus, intervening to mitigate oxidative stress may serve as a therapeutic strategy for managing DCM [4].

Alogliptin is an oral, antidiabetic treatment medication approved in many countries to treat patients with type 2 diabetes mellitus (T2DM) and has a generally favorable efficacy and safety profile [5]. Alogliptin, a DPP‐4 inhibitor, has been shown not to increase the incidence of major cardiovascular events, thereby reassuring clinicians that the drug can be used safely in patients with diabetes and recent acute coronary syndrome [6]. In an animal study, alogliptin alleviated ventricular hypertrophy, interstitial fibrosis, and diastolic dysfunction in diabetic rabbits [7]. However, no studies have investigated the application of alogliptin in DCM. Interestingly, alogliptin can not only decrease organ pathology and preserve mitochondrial function but also suppress oxidative stress in rodents fed a high‐fat diet [8]. These findings led to the hypothesis that alogliptin may affect DCM through oxidative stress.

Aurora kinase B (AURKB) is a member of a highly conserved family of serine/threonine kinases and plays a critical role in mitosis, cell cycle checkpoints, and normal physiology [9]. The deregulated AURKB repeated multiple enrichments in related items might be associated with the pathogenesis of DCM [10]. Furthermore, AURKB protects oocytes from oxidative stress [11]. Bioinformatics analysis revealed that AURKB is a target protein of alogliptin. Therefore, we hypothesized that alogliptin may affect DCM by regulating AURKB expression.

Our preliminary results demonstrated that AURKB was enriched at the neuroligin 2 (NLGN2) promoter and activated the transcription of NLGN2. The NLGN protein family consists of five members in humans (NLGN1, 2, 3, 4X, and 4Y) and four in mice (NLGN1, 2, 3, and 4), and regulates synaptic function [12]. Previous evidence has shown that NLGN2 is downregulated in rodents during hyperglycemia, hypoglycemia, and diabetic hypoglycemia, and that NLGN2 indirectly affects blood glucose levels by modulating insulin secretion [13]. Accordingly, we hypothesized a potential interaction between AURKB and NLGN2 in DCM.

Based on these findings, this study aimed to investigate the effect of alogliptin on DCM and the specific molecular mechanisms of action, which may involve AURKB and NLGN2 and their role in regulating oxidative stress.

## 2 Materials and Methods

### 2.1 Animal Models

C57BL/6J male mice (8 weeks old, 20–22 g, Aniphe Biolab, Nanjing, Jiangsu, China) were housed under a 12‐h light/12‐h dark cycle at a constant temperature with adequate food and water. The mice were assigned to seven groups of five mice each: normal, DCM, DCM + methylcellulose (MC), DCM + alogliptin, DCM + alogliptin + short hairpin RNA (sh)‐negative control (NC), DCM + alogliptin + sh‐AURKB, and DCM + alogliptin + sh‐NLGN2.

A DCM model in mice was constructed by intraperitoneal injection of 50 mg/kg of streptozotocin (STZ, HY‐13753, MedChemExpress, Monmouth Junction, NJ, USA) dissolved in 100 mM sodium citrate buffer (pH 4.5) for 5 days. The control mice were intraperitoneally injected with an equal volume of citrate buffer. One week after injection, mice with glucose levels > 300 mg/dL, as measured using a glucometer, were recognized as having diabetes. Four weeks before STZ injection, mice were injected with adeno‐associated virus AAV9 sh‐AURKB or sh‐NLGN2 (> 2 × 10^11^ GC/mL) [14].

Immediately after the successful establishment of the diabetes model, mice in the DCM group were treated with 45 mg/kg alogliptin (HPLC ≥ 98%, B34194, Yuanye Biotechnology, Shanghai, China), which was administered by gavage once a day. MC (0.5% concentration) is a frequently utilized agent in the evaluation of investigational agents for efficacy in preclinical models of disease and is considered a safe compound when administered orally [15]. Control mice were administered 0.5% MC (HY‐125861, MedChemExpress) via gavage for 10 weeks [16]. All relevant animal experiments were approved by the Animal Ethics Committee of the Second Hospital of Hebei Medical University (approval number: 2025‐AE297). The animal experiments were conducted in accordance with the ARRIVE guidelines.

One day before euthanasia, the mice were anesthetized, and echocardiography was performed. Blood samples were collected from mouse hearts using commercial tubes containing K3EDTA as an anticoagulant, and serum was collected by centrifugation at 1000 × *g* for 5 min using tubes without anticoagulant and stored at −20°C [17]. Ten weeks after alogliptin administration, the mice were weighed. According to the American Veterinary Medical Association (AVMA) Guidelines for the Euthanasia of Animals: 2020 Edition, euthanasia was performed by intraperitoneal injection of 100 mg/kg sodium pentobarbital. Mice exhibiting severe debilitation, sustained weight loss exceeding 20%, markedly reduced activity, matted fur, difficulty eating or drinking, or other irreversible signs of distress and stress responses were euthanized early and excluded from the study. Following euthanasia of all mice, animal death was confirmed by the cessation of heartbeat, respiration, and corneal reflexes. Heart samples were weighed to obtain heart weight (HW). A portion of the heart tissue was stored at −80°C, while the other portion was embedded in paraffin.

### 2.2 Echocardiography

The systolic and diastolic functions of the mouse heart were examined using noninvasive echocardiography (VEVO3100, Fujifilm Visualsonics, Toronto, ON, Canada) the day before the mice were sacrificed. Mice were anesthetized by intraperitoneal injection of 26 mg/kg propofol. The echocardiographic parameters included left ventricular fractional shortening (FS), ejection fraction (EF), left ventricular internal dimension–diastole (LVIDd), interventricular septal thickness at diastole (IVSD), and left ventricular posterior wall thickness dimensions (LVPWd).

### 2.3 Hematoxylin and Eosin (HE) Staining

Cardiac tissues were fixed in 10% neutral formalin buffer for 72 h, dehydrated with a gradient of ethanol, embedded in paraffin, and cut into 5 μm‐thick sections. The 5‐μm sections were dewaxed, hydrated, and stained with hematoxylin (H3136, Sigma‐Aldrich, St. Louis, MO, USA) for 3 min, rinsed with distilled water, stained with eosin (C0109; Beyotime, Shanghai, China) for 2 min, dried, and sealed. Finally, the sections were observed using a light microscope.

### 2.4 Masson Staining

Paraffin‐embedded sections of cardiac tissue were washed with 95% ethanol, stained with hematoxylin, washed with ethanol, picric acid solution, and acidic magenta solution, and stained with 1% phosphomolybdic acid (221856, Sigma‐Aldrich). Subsequently, the sections were treated with aniline blue stain (B8563, Sigma‐Aldrich) mixed with acetic acid and 1% acetic acid for 1 min. Finally, the sections were dehydrated in a gradient of ethanol, cleared in xylene, and sealed with neutral glue.

### 2.5 Wheat Germ Agglutinin (WGA) Staining Assay

Paraffin‐embedded sections of heart tissue were stained for cell membrane staining with wheat germ agglutinin and Alexa Fluor 488 (W11261, Thermo Fisher Scientific, Waltham, MA, USA). The sections were diluted in PBS at a ratio of 1:300 for 20 min, permeabilized with 0.1% Triton X‐100 (P0096‐100 mL, Beyotime) for 15 min, and observed under a fluorescence microscope.

### 2.6 Immunohistochemistry (IHC)

Cardiac tissue sections were deparaffinized for antigen retrieval with citrate buffer (pH 6.0) and treated with 3% H_2_O_2_. The sections were sealed overnight with 5% goat serum at 37°C for 30 min and probed with primary antibodies against collagen I (Col I, 1:100, ab270993, Abcam, Cambridge, MA, USA) and Col III (1:100, PA5‐99160, Thermo Fisher). Next, the sections were incubated with horseradish peroxidase (HRP)‐coupled secondary antibody (1:1000, G‐21234, Thermo Fisher) for 30 min at room temperature and detected using a DAB HRP color development kit (P0203, Beyotime). Hematoxylin staining was used for nuclear staining. The results were observed using a microscope. Image‐Pro Plus 6.0 software was used to analyze the images and calculate the positive staining rate.

### 2.7 TdT‐Mediated dUTP Nick‐End Labeling (TUNEL) Assay

Cardiomyocyte apoptosis in mouse heart tissues was detected using a One‐step TUNEL In Situ Apoptosis Kit (E‐CK‐A321, Elabscience Biotechnology Co. Ltd., Wuhan, Hubei, China). The paraffin sections were dewaxed, hydrated, and incubated with 100 μL of 1× proteinase K working solution at 37°C for 20 min. Mouse cardiomyocytes were collected, resuspended in PBS, fixed for 20 min at room temperature, and centrifuged at 600 × *g* for 5 min. Subsequently, 25 μL of the cell suspension was smeared on slides and placed in 0.2% Triton X‐100 for 10 min at 37°C. Next, 100 μL of TdT equilibration buffer was added dropwise to the prepared samples and incubated at 37°C for 10 min. Then, 50 μL of the labeling working solution was added dropwise for 60 min at 37°C in the dark. DAPI working solution was added for 5 min at room temperature, in the dark. Finally, the slides were sealed with a sealer containing an antifade mounting medium, and images were acquired using a microscope.

### 2.8 Cell Modeling

Mouse cardiomyocytes (CP‐M073, Procell) were cultured in mouse cardiomyocyte complete medium (CM‐M073, Procell) in an incubator containing 5% CO_2_ at 37°C. When the confluence of cardiomyocytes reached 50%–60%, the control group was cultured in DMEM with a glucose concentration of 5.55 mM with 24.5 mM mannitol added to maintain osmotic pressure, and the high‐glucose (HG) group was cultured in DMEM with a glucose concentration of 30 mM; both were incubated for 48 h [18].

Mouse cardiomyocytes were infected in the serum‐free medium containing sh‐AURKB or sh‐NLGN2, or overexpression (OE)‐AURKB lentiviruses, before HG treatment. The multiplicity of infection was 30 in the presence of 8 μg/mL polyglutamine. Two days after infection, the cells were screened with 2 μg/mL puromycin for 10 days before further culture for 3 generations [19].

After HG treatment, mouse cardiomyocytes were incubated in serum‐free medium for 24 h. Mouse cardiomyocytes were then treated with 50 μM alogliptin for 24 h. The controls were treated with an equal amount of DMSO for the same duration [8].

### 2.9 Reverse Transcription‐Quantitative Polymerase Chain Reaction (RT‐qPCR)

Total RNA was extracted from the heart tissue and mouse cardiomyocytes using the RNA isolator total RNA extraction reagent (R401‐01, Vazyme, Nanjing, Jiangsu, China). cDNA synthesis was performed using the PrimeScript RT Premix Kit (RR047Q, Takara, Dalian, Liaoning, China). Quantitative PCR was performed on an ABI 7500 RT‐PCR system (4351106, Thermo Fisher) using SYBR Green master mix (Q111‐02, Vazyme). Expression was calculated using the 2^−ΔΔCT^ formula with GAPDH as the internal reference. Primer sequences are listed in Table 1.

**TABLE 1:** Primer sequences for RT‐qPCR.

Gene name	Forward sequence (5′‐3′)	Reverse sequence (5′‐3′)
AURKB	CTTCTACGACCAGCAGAGGATC	GGCATCTGACAGTTCCTCCATG
NLGN2	CGATGTCATGCTCAGCGCAGTA	CCACACTACCTCTTCAAAGCGG
GAPDH	CATCACTGCCACCCAGAAGACTG	ATGCCAGTGAGCTTCCCGTTCAG

Abbreviations: AURKB, aurora kinase B; GAPDH, glyceraldehyde‐3‐phosphate dehydrogenase; NLGN2, neuroligin 2.

### 2.10 Western Blot (WB)

Total protein content was determined in heart tissue and mouse cardiomyocytes using lysis buffer (P0013B, Beyotime) containing 1% phenylmethylsulfonyl fluoride (ST506, Beyotime). Proteins were separated using SDS‐PAGE and transferred to PVDF membranes. The membranes were sealed with 5% skimmed milk and incubated overnight with primary antibodies against AURKB (1:500, A21918, ABclonal, Wuhan, Hubei, China), NLGN2 (1:500, A14289, ABclonal), β‐actin (1:10000, AC038, ABclonal), and with secondary antibody goat anti‐rabbit coupled to HRP (1:5000, G‐21234, Thermo Fisher). Blots were visualized using an ECL kit.

### 2.11 Creatine Kinase‐MB (CK‐MB) and Lactate Dehydrogenase (LDH) Measurement

CK‐MB and LDH levels in mouse serum were detected using CK‐MB (E006‐1‐1, Nanjing Jiancheng Bioengineering Institute, Nanjing, Jiangsu, China) and LDH (A020‐1‐2, Nanjing Jiancheng) kits. For the determination of CK‐MB, mouse serum samples were mixed with Reagents I and II at 37°C for 2 min, and the absorbance (A1) was immediately measured at 340 nm. Three minutes later, the absorbance (A2) was measured. ΔA = A2‐A1. F = [total reaction volume (mL) × 1000/sample volume (mL) × 6.22 × cuvette aperture (cm)] × 2. CK‐MB viability (U/L) = ΔA/min × F.

For LDH measurements, serum samples were mixed with matrix buffer and coenzyme I at 37°C for 15 min and then mixed with 2,4‐dinitrophenylhydrazine at 37°C for 15 min. The samples were treated with NaOH solution for 3 min, and the absorbance was measured at 440 nm. Serum LDH activity (U/L) = (A assay − A control)/(A standard − A blank) × (C standard × V standard) × (1000 / V sample) × N, where C standard is the concentration of standard, V standard is the amount of standard added (mL), V sample is the amount of sample added (mL), and N is the number of times the sample was diluted before testing.

### 2.12 Reactive Oxygen Species (ROS) Measurement

ROS levels in mouse cardiac tissue were measured using the ROS Fluorometric Assay Kit (Green) (E‐BC‐K138‐F; Elabscience). According to the manufacturer's protocol, cardiac tissues were placed in pre‐cooled PBS, cut into small pieces of approximately 1 mm^3^ with ophthalmic scissors, and incubated with an enzyme digestive solution in a water bath for 30 min at 37°C. Serum‐containing medium was then added and filtered through a 300‐mesh nylon mesh, and the filtered cells were collected and centrifuged at 500 × *g* for 10 min. A single‐cell suspension was prepared by resuspension in PBS. Reagent I working solution was then added to the single‐cell suspension and incubated at 37°C for 1 h under light protection. After centrifugation at 1000 × *g* for 10 min, the cells were resuspended in serum‐free cell culture medium and placed in a microplate reader for detection.

Mouse cardiomyocytes were seeded in confocal dishes at 37°C, and 5 μM CM‐H2DCFA (C6827, Thermo Fisher) was added in a dark environment and incubated for 10 min before observation under a fluorescence microscope.

### 2.13 Measurement of Malondialdehyde (MDA) Levels and Superoxide Dismutase (SOD) Activity

MDA content and SOD activity were determined using an MDA content assay kit (BC0025, Solarbio, Beijing, China) and an SOD assay kit (S0101S, Beyotime). For MDA determination, mouse cardiomyocytes were ultrasonically fragmented with the extraction solution and centrifuged at 8000 × *g* for 10 min at 4°C. The supernatant was added to a centrifuge tube and heated with 0.3 mL of MDA assay working solution in boiling water for 60 min. After cooling, the samples were centrifuged at 1000 × *g* for 10 min, and the absorbance was measured at 532 and 600 nm.

For SOD determination, an SOD sample preparation solution was added to fully lyse the mouse cardiomyocytes. The samples were then added to the WST‐8/enzyme working solution and reaction initiation solution sequentially and incubated at 37°C for 30 min. Finally, the absorbance was measured at 450 nm.

### 2.14 Cell Counting Kit‐8 (CCK‐8)

Mouse cardiomyocyte viability was assessed using a CCK‐8 kit (C0037; Beyotime). Mouse cardiomyocytes from different groups were seeded in 96‐well plates at 5 × 10^3^ cells/well, added with 25 mM glucose, treated with 0.1 μM alogliptin, and incubated at 37°C for 24 h. Subsequently, 10 μL of CCK‐8 reagent was added to each well and incubated at 37°C for 2 h. The absorbance was measured at 450 nm.

### 2.15 Dual‐Luciferase Assay

Mouse cardiomyocytes were transfected after the sequence of NLGN2 was inserted into the pGL3‐basic plasmid (HG‐VQP0121, HonorGene, Changsha, Hunan, China) and the Renilla vector pRL‐TK (VT1568, YouBio, Changsha, Hunan, China). Successfully transfected cardiomyocytes were collected and assayed using a dual‐luciferase reporter assay kit (DL101‐01; Vazyme). Relative luciferase activity was expressed as the ratio of firefly luciferase activity to Renilla luciferase activity, with the Renilla luciferase fluorescence value used as a reference.

### 2.16 Chromatin Immunoprecipitation (ChIP)

As per the instructions provided with the ChIP assay kit (P2078, Beyotime), 1 × 10^7^ cellular chromatin samples were sonicated to generate 200–400 bp fragments in ChIP dilution buffer. DNA fragments were immunoprecipitated in dilution buffer using anti‐AURKB (1:100, 3094, Cell Signaling, Beverly, MA, USA) or anti‐IgG (1:100, 30,000–0‐AP, Proteintech, Wuhan, Hubei, China) antibodies, followed by overnight incubation at 4°C. Antibody complexes were enriched using immunomagnetic beads and crosslinked, and DNA fragments were released in an elution buffer at 65°C for 5 h. qPCR was conducted using promoter‐specific primer sequences for NLGN2.

### 2.17 Statistical Analysis

Data are presented as mean ± standard error of the mean and were processed using Prism 10.4.2 (GraphPad Software Inc., San Diego, CA, USA). One‐factor pairwise comparisons were made using the t‐test, and multi‐group comparisons were made using one‐way or two‐way ANOVA. The Tukey test was used for post hoc analysis. The significance level was set at *p* < 0.05. Animal experiments were performed using five mice per group, and cellular experiments were independently repeated three times for each group.

## 3 Results

### 3.1 Alogliptin Ameliorates the Cardiac Dysfunction of Mice Induced by STZ

**FIGURE 1:**
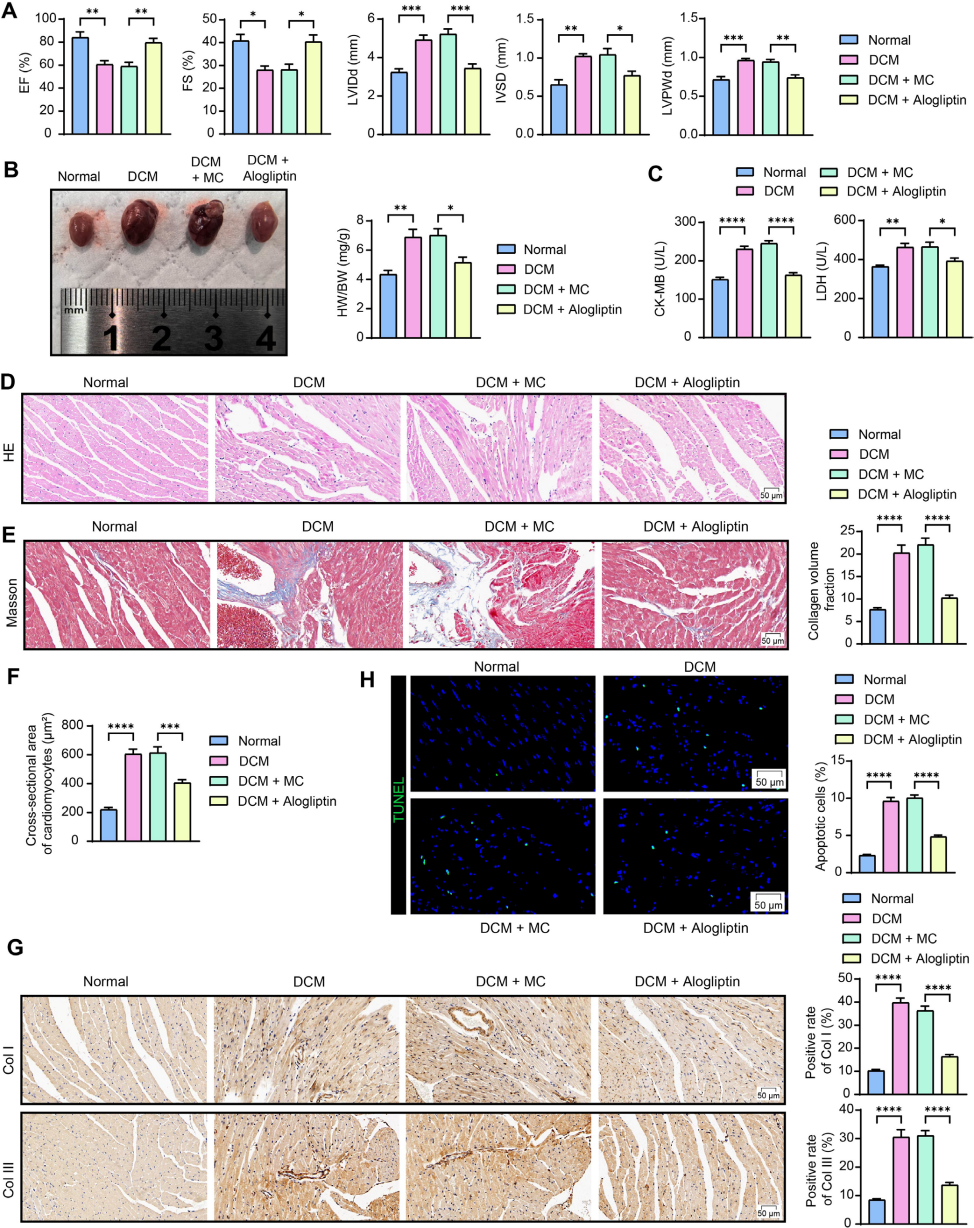
Alogliptin relieves DCM in mice. (A) Measurement of EF, FS, LVIDd, IVSD, and LVPWd. (B) Representative pictures of mouse hearts and HW/BW ratios. (C) Detection of serum levels of CK‐MB and LDH in mice. (D) HE staining of cardiac lesions in mouse heart tissue. (E) Masson staining of collagen fibrillation in mouse heart tissue. (F) WGA staining of the cross‐sectional area of cardiomyocytes in mouse heart tissue. (G) IHC staining of cardiomyocytes in mouse heart tissue. (H) IHC staining to observe and quantitatively analyze Col I and Col III contents. (H) TUNEL to analyze apoptosis of cardiomyocytes in mouse heart tissue. Animal experiments were performed with five mice per group. One‐way ANOVA was used for single‐factor comparisons among multiple groups. *p < 0.05, **p < 0.01, ***p < 0.001, ****p < 0.0001.

Echocardiography (Figures 1A and S1A) revealed diastolic and systolic dysfunction in the hearts of mice in the DCM group, with decreased EF and FS and increased LVIDd, IVSD, and LVPWd. However, treatment with alogliptin ameliorated cardiac dysfunction in mice and reversed the trends of EF, FS, LVIDd, IVSD, and LVPWd in the DCM group. The hearts of mice in the DCM group were more hypertrophic than those in the normal group; alogliptin treatment alleviated the symptoms of cardiac hypertrophy in mice, which was confirmed by the HW/BW ratio results (Figure 1B). The serum levels of both CK‐MB and LDH were increased in the sera of DCMe mice, and this trend was reversed by alogliptin treatment (Figure 1C). HE staining (Figure 1D) revealed that the myocardial structure in DCM mice was disrupted, and alogliptin treatment alleviated the myocardial damage caused by DCM. Masson staining (Figure 1E) revealed that the collagen volume fraction was increased in the cardiac tissues of DCM mice but decreased after alogliptin treatment. WGA staining (Figure 1F) revealed that the cross‐sectional area of cardiomyocytes increased in the DCM group but decreased after alogliptin treatment. IHC staining (Figure 1G) showed that Col I and Col III levels were increased in the DCM group but decreased after alogliptin treatment. TUNEL (Figure 1H) showed that the apoptotic rate of cardiomyocytes in the cardiac tissues of DCM mice was significantly higher than that in the normal group. In contrast, alogliptin treatment reduced the cardiomyocyte apoptotic rate.

### 3.2 Alogliptin Enhances AURKB Expression in HG‐induced Cardiomyocytes

**FIGURE 2:**
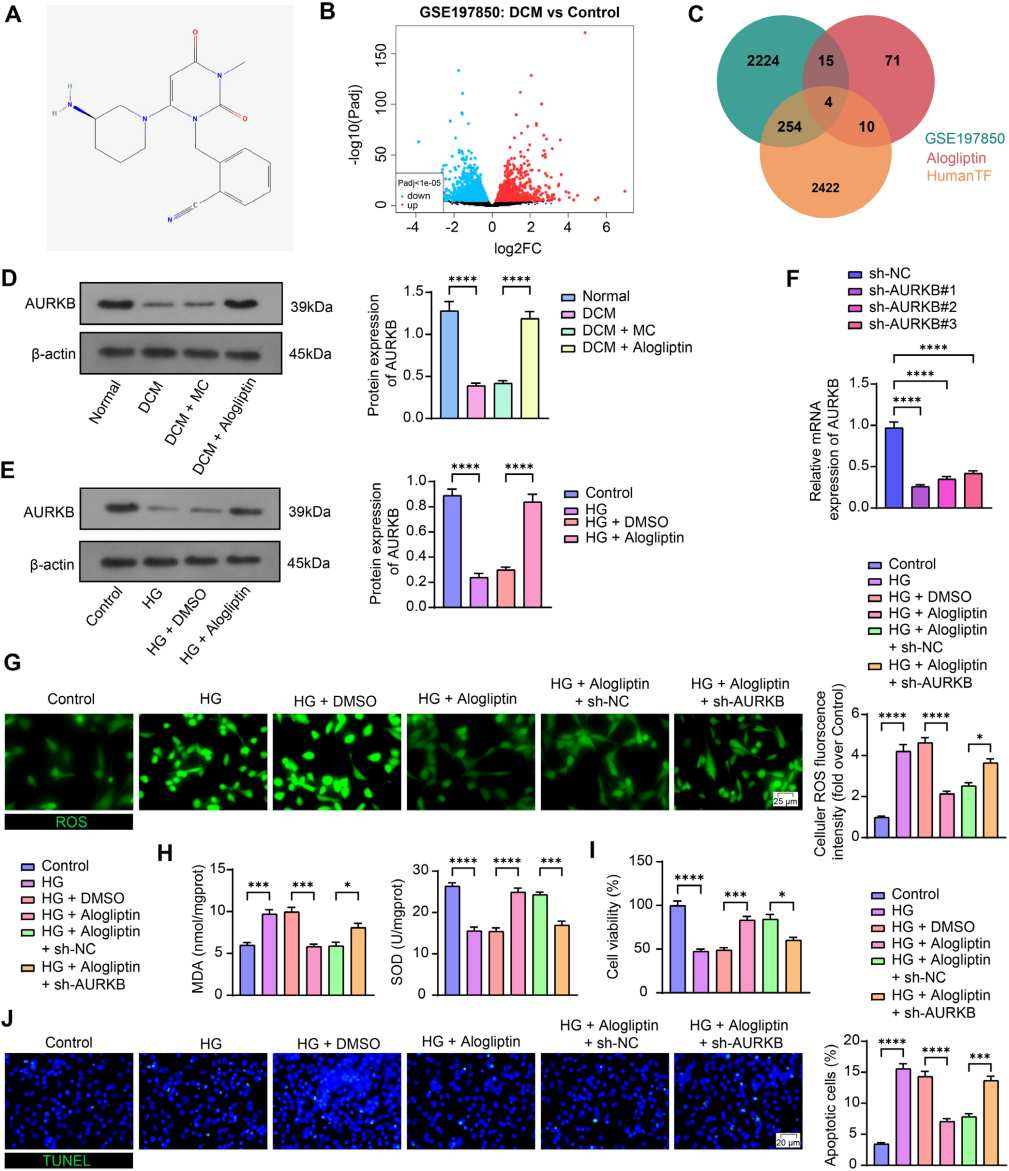
Alogliptin ameliorates DCM by promoting the expression of AURKB. (A) Chemical structure of alogliptin. (B) Analysis of DEGs between DCM and Control cardiomyocytes in the GSE197850 dataset. (C) The intersection of downstream targets of alogliptin, DEGs in the GSE197850 dataset, and human transcription factor and co‐factor list. (D) WB validation of AURKB protein expression in mouse heart tissues. (E) WB detection of AURKB protein expression in mouse cardiomyocytes. (F) RT‐qPCR validation of the knockdown efficiency of AURKB. (G) ROS levels in mouse cardiomyocytes. (H) Kits to detect MDA levels and SOD activity in the supernatant of mouse cardiomyocytes. (I) CCK‐8 assay to detect the viability of mouse cardiomyocytes. (J) TUNEL to detect the apoptosis rate of mouse cardiomyocytes. The cellular experiments were repeated three times independently for each group. One‐way ANOVA was used for comparison between single‐factor multiple groups. *p < 0.05, ***p < 0.001, ****p < 0.0001.

SwissTargetPrediction (http://swisstargetprediction.ch/) was utilized to predict the downstream targets of alogliptin based on the chemical structure of alogliptin obtained from PubChem Substance (https://www.ncbi.nlm.nih.gov/pcsubstance/?term=) (Figure 2A). Then, differentially expressed genes (DEGs) between diabetic cardiomyocytes and control cardiomyocytes were analyzed in the GSE197850 dataset from the GEO database. From HumanTFDB (http://bioinfo.life.hust.edu.cn/HumanTFDB#!/), the human transcription factors and cofactors were downloaded and intersected with DEGs in the GSE197850 dataset (adj. *p* < 1e^−5^) (Figure 2B) and downstream targets of alogliptin on Jvenn (https://jvenn.toulouse.inrae.fr/app/example.html). Four intersections (Figure 2C) were obtained: AURKB (Log_2_FoldChange = −1.3543655), BRD4 [20], CDK1 (Log_2_FoldChange = −0.742076), and RXRA [21]. After excluding the genes already reported, AURKB with the most significant low expression was selected for further study.

WB (Figure 2D) assay revealed that protein expression of AURKB was reduced in mouse heart tissues in the DCM group, whereas alogliptin upregulated AURKB expression. HG‐induced mouse cardiomyocytes were selected for subsequent experiments and treated with 50 μM alogliptin. WB (Figure 2E) revealed that HG treatment downregulated AURKB expression; alogliptin upregulated AURKB expression. Three shRNAs (sh‐AURKB#1, sh‐AURKB#2, sh‐AURKB#3) were designed to knock down AURKB expression in mouse cardiomyocytes. RT‐qPCR (Figure 2F) verified that all of them decreased AURKB expression, and sh‐AURKB#1 was the most efficient. Therefore, we chose sh‐AURKB#1 for subsequent experiments. The CM‐H2DCFA probe labeling assay (Figure 2G) showed that HG treatment upregulated ROS levels in mouse cardiomyocytes. Alogliptin treatment downregulated ROS levels, while further knockdown of AURKB upregulated ROS levels. In mouse cardiomyocyte supernatants (Figure 2H), HG treatment upregulated MDA levels and diminished SOD activity. However, MDA levels decreased, and SOD activity increased after alogliptin treatment. Combined knockdown of AURKB reversed the trend after alogliptin treatment. CCK‐8 (Figure 2I) results evinced that HG treatment decreased mouse cardiomyocyte viability. Alogliptin treatment restored mouse cardiomyocyte viability, and combined knockdown of AURKB again decreased mouse cardiomyocyte viability. TUNEL (Figure 2J) results noted that HG treatment increased the apoptosis rate of mouse cardiomyocytes. Alogliptin treatment decreased the apoptosis rate, while the combined knockdown of AURKB again upregulated the apoptosis rate of mouse cardiomyocytes.

### 3.3 AURKB Knockdown Reverses the Attenuating Effect of Alogliptin on DCM in Mice

**FIGURE 3:**
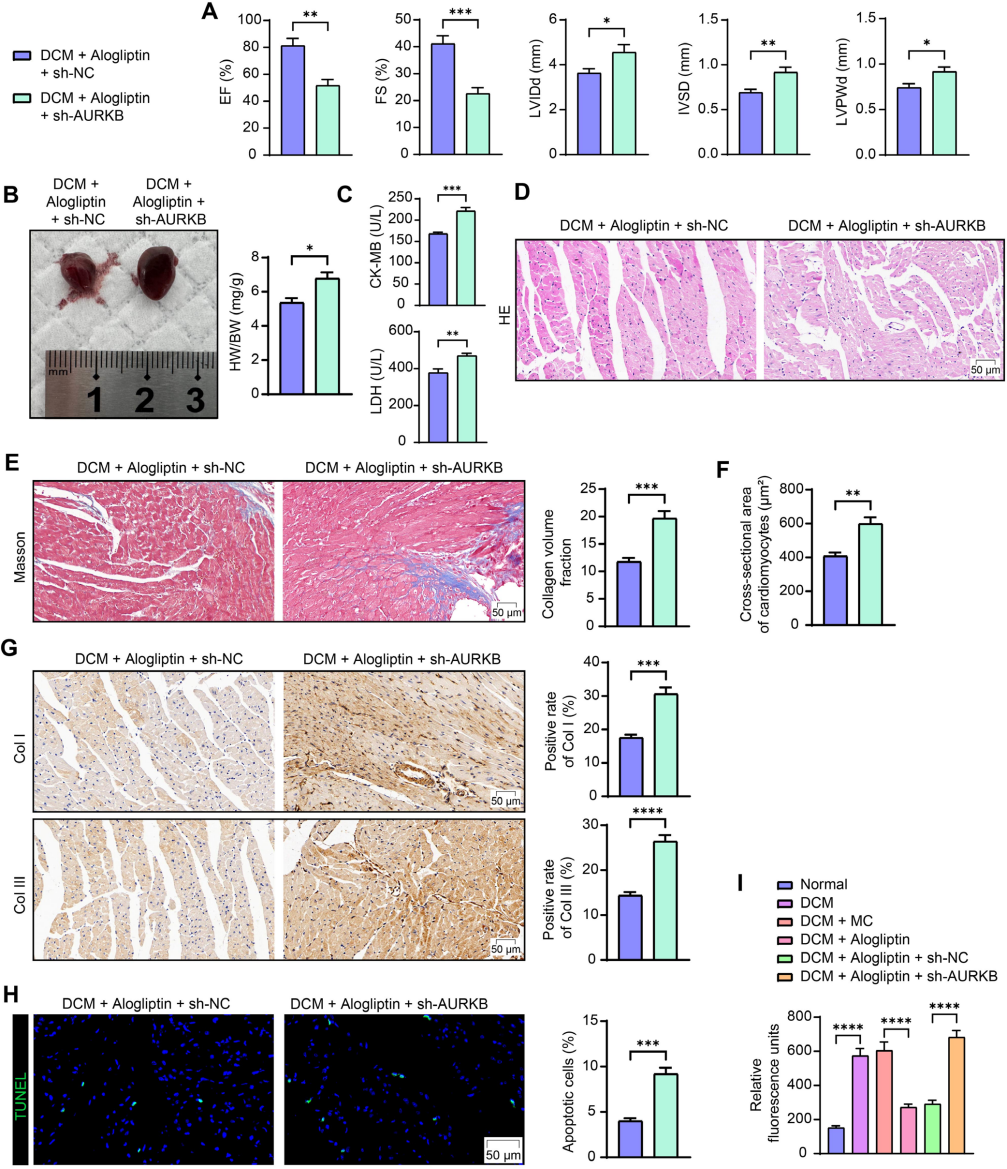
AURKB knockdown reverses the protective effect of alogliptin on DCM in mice. (A) Measurement of EF, FS, LVIDd, IVSD, and LVPWd. (B) Representative pictures of mouse hearts and HW/BW ratios. (C) Detection of serum levels of CK‐MB and LDH in mice. (D) HE staining of cardiac lesions in mouse hearts. (E) Masson staining of collagen fibrillation in mouse heart tissue. (F) WGA staining of the cross‐sectional area of cardiomyocytes in mouse heart tissue. (G) IHC staining of Col I and Col III contents in mouse heart tissues. (H) TUNEL to analyze apoptosis of cardiomyocytes in mouse heart tissues. (I) ROS levels in mouse heart tissues. Animal experiments were performed with five mice per group. The *t*‐test was used for single‐factor comparisons between two groups, and one‐way ANOVA was used for single‐factor comparisons among multiple groups. *p < 0.05, **p < 0.01, ***p < 0.001, ****p < 0.0001.

Echocardiography (Figures 3A and S1B) revealed that combined knockdown of AURKB reintroduced cardiac diastolic and systolic dysfunction in mice, decreased EF and FS, and increased LVIDd, IVSD, and LVPWd. The hearts of mice in the DCM + alogliptin + sh‐AURKB group were more hypertrophic than those in the control group, along with higher HW/BW ratios (Figure 3B). AURKB knockdown upregulated the serum levels of CK‐MB and LDH in mice (Figure 3C). HE staining (Figure 3D) revealed that AURKB knockdown disrupted the myocardial structure after alogliptin treatment. Masson staining (Figure 3E) showed that the collagen volume fraction increased in the cardiac tissues after AURKB knockdown. WGA staining (Figure 3F) revealed that the cross‐sectional area of cardiomyocytes in mice was increased after AURKB knockdown. IHC (Figure 3G) revealed that AURKB knockdown elevated Col I and Col III contents. These results proved obvious interstitial fibrosis in the cardiac tissues of mice in the DCM + alogliptin + sh‐AURKB group. TUNEL (Figure 3H) revealed that AURKB knockdown upregulated apoptosis of cardiomyocytes in the cardiac tissues of mice. Increased levels of ROS in the cardiac tissues of DCM mice were reversed by alogliptin. AURKB knockdown upregulated ROS levels (Figure 3I).

### 3.4 AURKB Activates the Transcription of NLGN2

**FIGURE 4:**
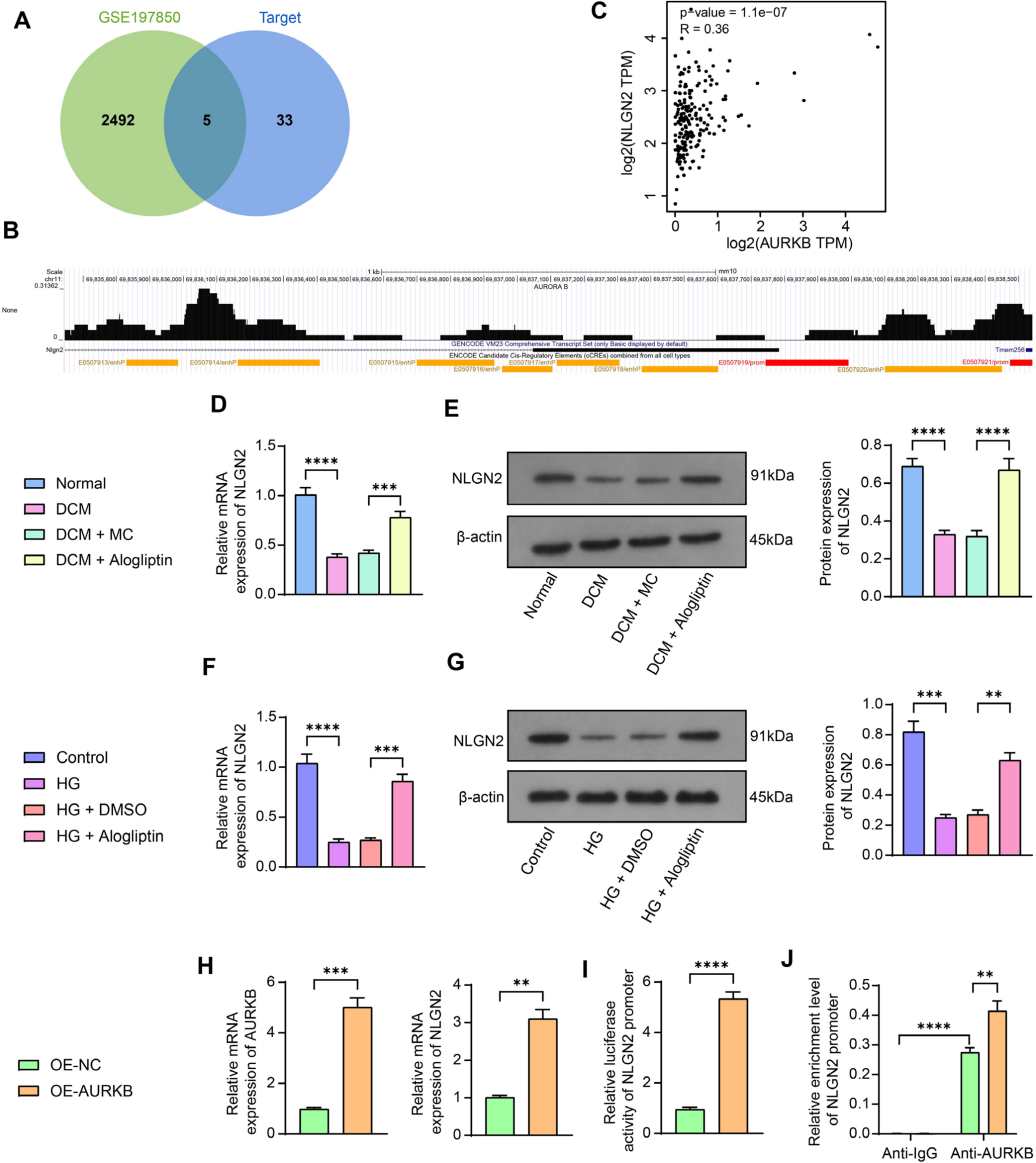
AURKB transcriptionally regulates NLGN2 expression. (A) Intersection of downstream targets of AURKB and DEGs in the GSE197850 dataset. (B) ChIP dataset analyzed the binding of AURKB to the NLGN2 promoter. (C) GEPIA analyzed the expression relationship between AURKB and NLGN2. (D) RT‐qPCR detected NLGN2 expression in mouse cardiac tissues. (E) WB detected the protein expression of NLGN2 in mouse cardiac tissues. (F) RT‐qPCR detection of NLGN2 expression in mouse cardiomyocytes. (G) WB detection of NLGN2 protein expression in mouse cardiomyocytes. (H) RT‐qPCR detection of mRNA expression of AURKB and NLGN2 in mouse cardiomyocytes. (I) Dual‐luciferase assay for detection of NLGN2 luciferase activity in mouse cardiomyocytes. (J) ChIP assay to verify the binding of AURKB to the NLGN2 promoter in mouse cardiomyocytes. The cellular experiments were repeated three times independently for each group. Single‐factor comparisons were made between two groups using the *t*‐test, and one‐way/two‐way ANOVA was used for multigroup comparisons. **p < 0.01, ***p < 0.001, ****p < 0.0001, ns indicates no significant difference.

A set of gene targets with a total score = 604.73 with AURKB was downloaded from GeneCards (https://www.genecards.org/) and then intersected with DEGs in the GSE197850 dataset (adj. *p* < 1e^−5^) by Jvenn. Five intersections were obtained (Figure 4A): AURKB, CYB5D1 (Log_2_FoldChange = 0.5044714), NLGN2 (Log_2_FoldChange = −0.8424253), EIF4A1 (Log_2_FoldChange = 0.4255716), and PER1 (Log_2_FoldChange = 0.4941806). The only intersecting gene with significantly downregulated expression, NLGN2, was selected for the study. ChIP‐Seq analysis in UCSC (https://genome.ucsc.edu/cgi‐bin/hgGateway) revealed that AURKB had a binding peak in the promoter region of NLGN2 (Figure 4B). GEPIA (http://gepia.cancer‐pku.cn/index.html) analysis revealed that AURKB was positively correlated with NLGN2 expression in Heart–Left Ventricle (Figure 4C).

RT‐qPCR (Figure 4D) and WB (Figure 4E) assays revealed that NLGN2 was reduced in the cardiac tissue of DCM mice. HG treatment downregulated NLGN2 levels in mouse cardiomyocytes (Figure 4F,G). After successful overexpression of AURKB in mouse cardiomyocytes (Figure 4H), NLGN2 mRNA expression was increased as well. Dual‐luciferase assay (Figure 4I) manifested that AURKB overexpression enhanced the luciferase activity of the NLGN2 promoter. ChIP assay (Figure 4J) showed that AURKB was enriched at the NLGN2 promoter, and AURKB overexpression elevated anti‐AURKB enrichment at the NLGN2 promoter.

### 3.5 NLGN2 Knockdown Reverses the Attenuating Effect of Alogliptin on HG‐exposed Cardiomyocytes

**FIGURE 5:**
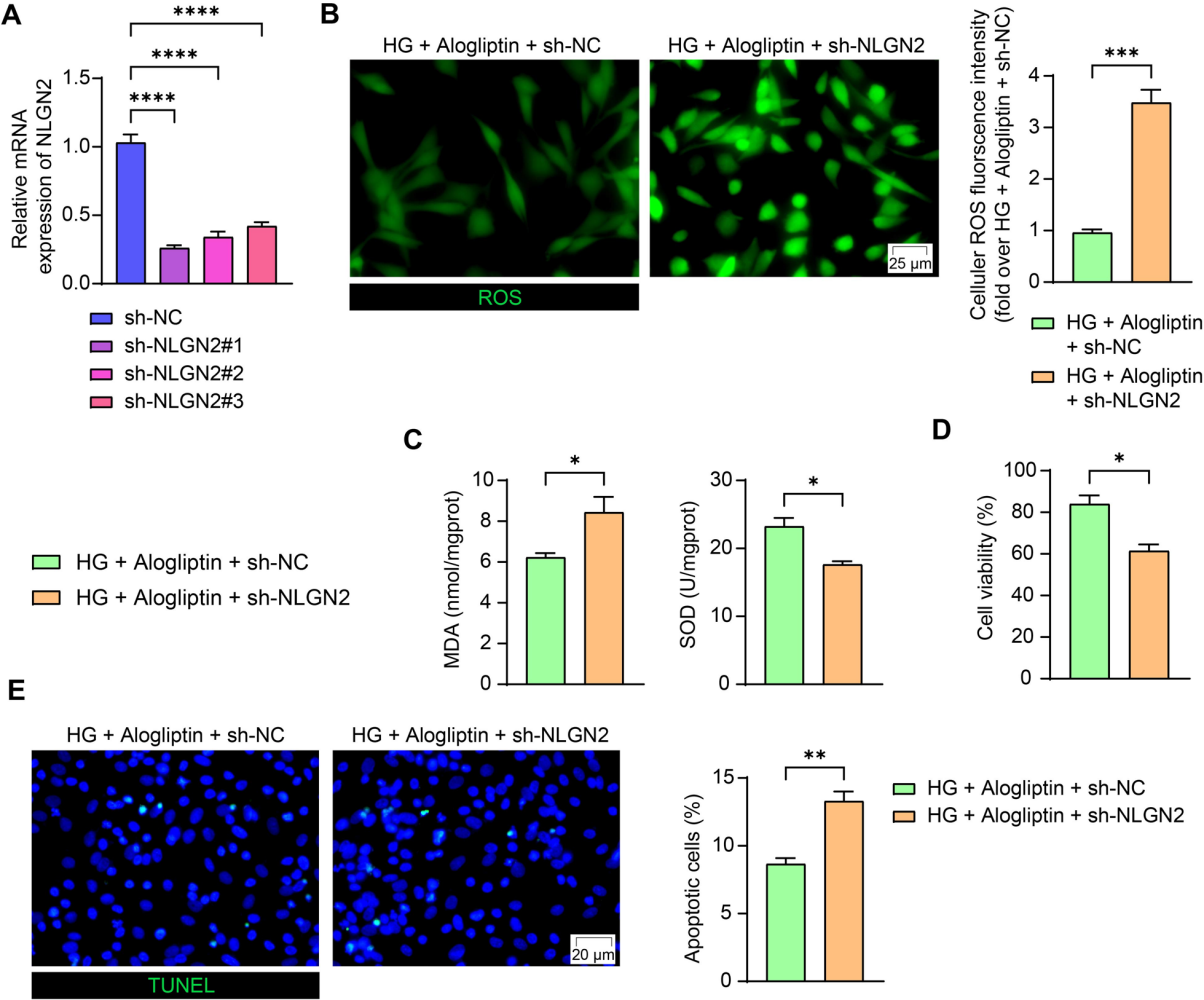
Combined knockdown of NLGN2 reverses the attenuation of oxidative stress by alogliptin in HG‐exposed cardiomyocytes. (A) RT‐qPCR to verify the knockdown efficiency of NLGN2. (B) CM‐H2DCFA probe labeling assay to detect ROS levels in mouse cardiomyocytes. (C) Kit detection of MDA levels and SOD activity in mouse cardiomyocyte supernatant. (D) CCK‐8 assay to detect the viability of mouse cardiomyocytes. (E) TUNEL to detect the apoptosis rate of mouse cardiomyocytes. The cellular experiments were repeated three times independently in each group. Single‐factor comparisons were made between two groups using the t‐test, and one‐way ANOVA was used for multigroup comparisons. *p < 0.05, **p < 0.01, ***p < 0.001, ****p < 0.0001.

Three shRNAs were designed to knock down NLGN2 (sh‐NLGN2#1, sh‐NLGN2#2, and sh‐NLGN2#3). RT‐qPCR (Figure 5A) showed that sh‐NLGN2#1 had the best knockdown efficiency. Therefore, it was selected for subsequent experiments. CM‐H2DCFA probe labeling assay (Figure 5B) noted that NLGN2 knockdown upregulated ROS levels in mouse cardiomyocytes. NLGN2 knockdown upregulated MDA levels and downregulated SOD activity (Figure 5C). CCK‐8 assay (Figure 5D) noted that NLGN2 knockdown decreased the viability of mouse cardiomyocytes. TUNEL (Figure 5E) showed that NLGN2 knockdown upregulated apoptosis of mouse cardiomyocytes.

### 3.6 Alogliptin Ameliorates DCM via the AURKB/NLGN2 Signaling

**FIGURE 6:**
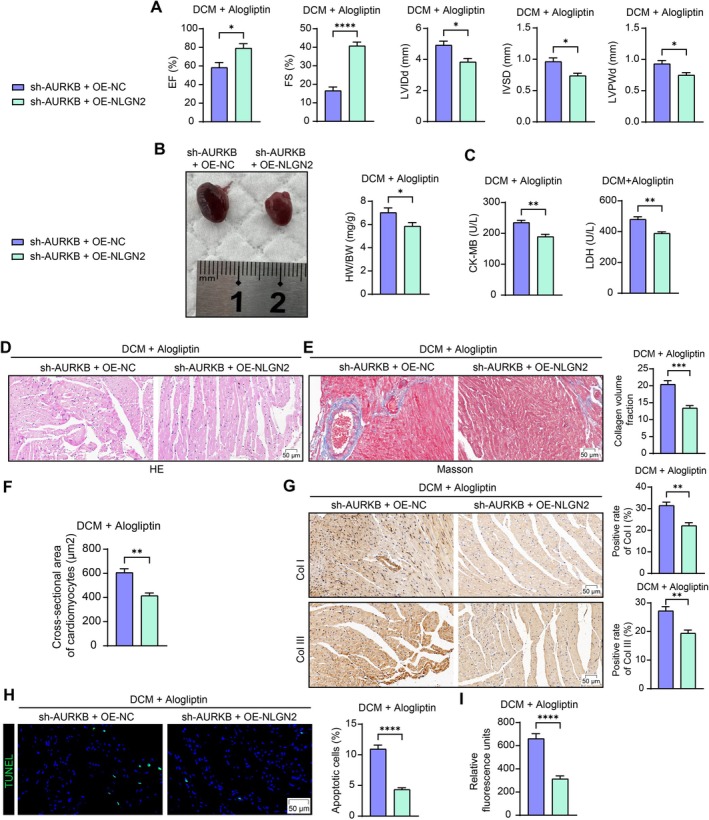
Alogliptin ameliorates DCM by modulating the AURKB/NLGN2 signaling. (A) Measurement of EF, FS, LVIDd, IVSD, and LVPWd. (B) Representative pictures of mouse hearts and HW/BW ratios. (C) Detection of serum levels of CK‐MB and LDH in mice. (D) HE staining of cardiac lesions. (E) Masson staining of collagen fibrillation in mouse heart tissue. (F) WGA staining of the cross‐sectional area of cardiomyocytes in mouse heart tissue. (G) IHC staining of Col I and Col III contents in mouse heart tissues. (H) TUNEL to analyze apoptosis of cardiomyocytes in mouse heart tissue. (I) Relative fluorescence units of ROS in mouse heart tissues. Animal experiments were performed with five mice per group. The t‐test was used for single‐factor comparisons between the two groups. *p < 0.05, **p < 0.01, ***p < 0.001, ****p < 0.0001.

NLGN2 overexpression attenuated cardiac diastolic and systolic dysfunction, increased EF and FS, and decreased LVIDd, IVSD, and LVPWd in mice (Figures 6A and S1C), downregulated the HW/BW ratio (Figure 6B), reduced the serum levels of CK‐MB and LDH (Figure 6C) in mice, and improved myocardial damage (Figure 6D). Further staining results revealed that after NLGN2 overexpression, collagen volume fraction was reduced in cardiac tissues of mice (Figure 6E), the cross‐sectional area of cardiomyocytes was reduced (Figure 6F), and Col I and Col III contents were diminished (Figure 6G), indicating improvement in interstitial fibrosis in the heart tissue of mice. Finally, NLGN2 overexpression decreased apoptosis of cardiomyocytes (Figure 6H) and ROS levels (Figure 6I) in the heart tissue of mice.

## 4 Discussion

DCM is a critical complication that poses a significant threat to the health of patients with diabetes, for which there are no specific drugs targeting the pathological mechanism [22]. DPP‐4 inhibitors are glucose‐lowering medications for T2DM, and evidence suggests that diabetes drugs targeting the enzyme DPP‐4 can improve heart health [17]. Alogliptin is representative of DPP‐4 inhibitors and shows favorable safety and efficacy for T2DM [23]. This study hereby probed into the potential effect of alogliptin on DCM.

DCM typically manifests as myocardial fibrosis, diastolic dysfunction, systolic dysfunction, increases in oxidative stress, and clinical heart failure [24]. In DCM, significant myofiber disruption and increased collagen deposition are observed, and cardiac damage is demonstrated by increased circulating levels of CK‐MB and LDH [25]. Our results indicated that alogliptin could reverse the trend of EF, FS, LVIDd, and LVPWd, attenuate cardiac hypertrophy, interstitial fibrosis, and cardiomyocyte apoptosis, and decrease serum CK‐MB and LDH levels and Col I and Col III contents, thus alleviating DCM. Alogliptin improved postprandial endothelial dysfunction, coronary flow reserve, and glycemic control in T2DM patients, with high efficacy and good tolerance [26]. Alogliptin alleviated interstitial fibrosis, ventricular hypertrophy, and diastolic dysfunction, and reduced oxidative stress in diabetic rabbits [7]. Numerous articles have proved the therapeutic effects of alogliptin on T2DM, and our study provides further support for its efficacy in DCM.

Our results demonstrated the decline in AURKB protein expression in DCM mice, and HG‐downregulated AURKB in mouse cardiomyocytes was elevated by alogliptin. AURKB is a serine/threonine kinase that plays a pivotal role in the regulation of cell division and mitosis, particularly in the separation of chromosomes [27]. ROS production in cardiomyocytes is a vicious circle, leading to post‐translational modification of proteins, as well as inflammation, cardiac hypertrophy, and fibrosis, ultimately causing cell death and cardiac dysfunction [28]. Our results unraveled that alogliptin could reduce levels of ROS and MDA and increase SOD activity, whereas its combined use with AURKB downregulation resulted in the opposite results in HG‐induced cardiomyocytes. Previous results demonstrated the function of AURKB in preserving the female reproductive lifespan by protecting oocytes from oxidative stress [11]. Consistently, the in vivo experiments illustrated that alogliptin combined with silencing of AURKB caused cardiac systolic and diastolic dysfunction, cardiac hypertrophy, interstitial fibrosis, and altered the tendency of LVIDd, IVSD, LVPWd, serum CK‐MB and LDH levels, and Col I and Col III contents in DCM mice. Furthermore, alogliptin could restore cardiomyocyte viability and decrease cardiomyocyte apoptosis, while AURKB knockdown led to contrary observations. Taken together, alogliptin could enhance AURKB expression in DCM, and silencing AURKB could abrogate the ameliorating function of alogliptin in DCM.

AURKB may be hypermethylated and reduced in DCM to alter the expression of other important cardiac genes via intermediate phosphorylation targets [29]. AURKB promotes tumorigenesis in different cancers by epigenetically activating the downstream targets [30, 31]. We observed decreased NLGN2 expression in the cardiac tissues of DCM mice, and AURKB overexpression increased the enrichment of anti‐AURKB at the NLGN2 promoter region. NLGN3 pretreatment can alleviate H_2_O_2_‐induced ROS production and oxidative injury in cultured osteoblasts [32]. Our results revealed that NLGN2 knockdown upregulated MDA and ROS levels and downregulated SOD activity, reduced the viability of HG‐induced cardiomyocytes, and accelerated cardiomyocyte apoptosis. A previous study has discovered a connection between oxidative stress and synaptic deficits in schizophrenia mediated by NLGN2 [33]. The animal experiments further indicated that NLGN2 overexpression alleviated the pathological conditions of DCM mice in the presence of AURKB knockdown. Collectively, alogliptin could improve DCM by modulating the AURKB/NLGN2 signaling.

A potential shortcoming of this study is the exclusive utilization of male mice in the experimental model. Given the established sex disparities in the prevalence and progression of DCM [34], it is possible that our findings may not entirely mirror the disease course observed in females. Future studies incorporating both sexes will be essential for the validation and extension of these findings.

## 5 Conclusion

**FIGURE 7:**
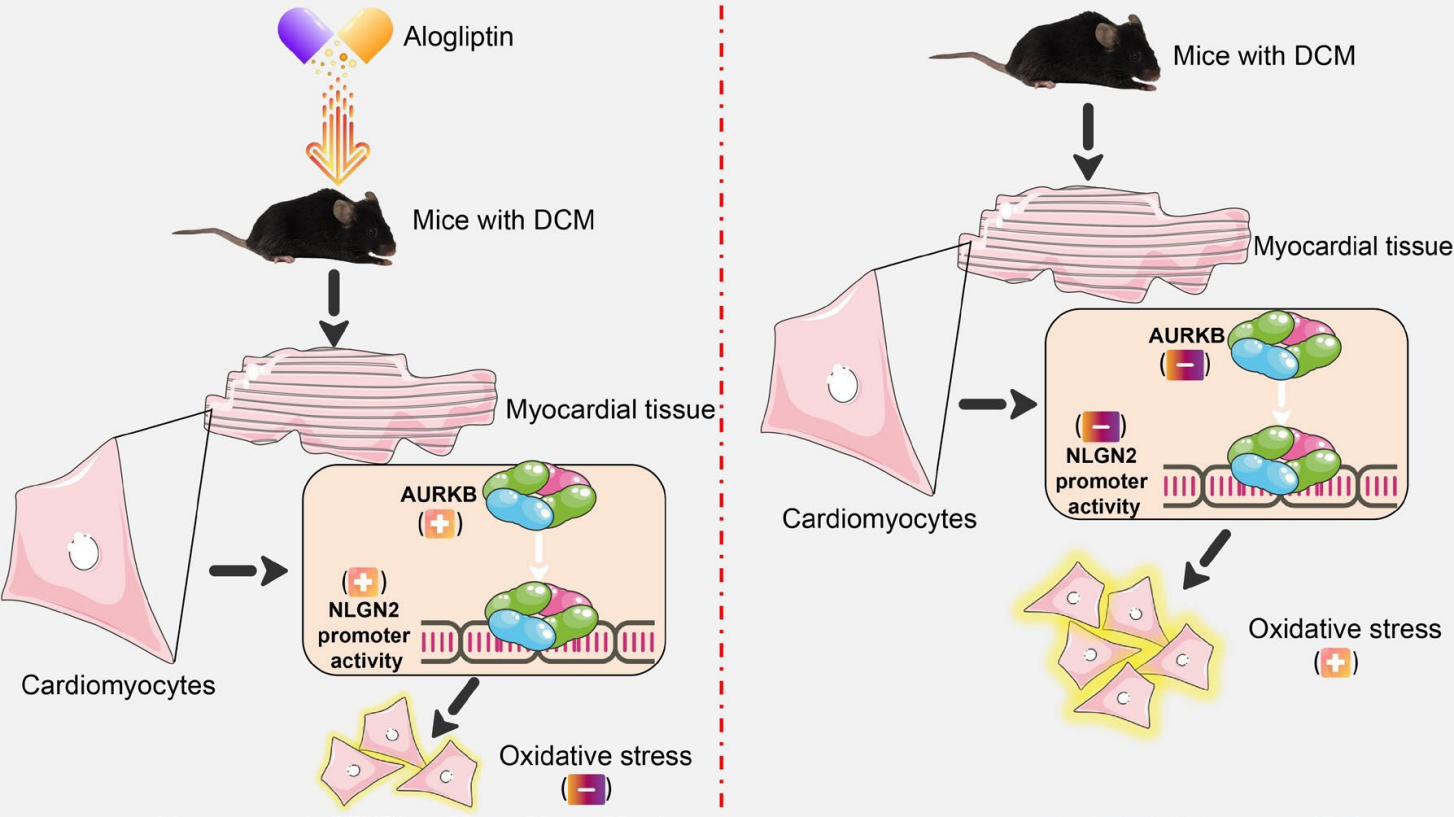
The proposed signaling cascade of this study. Alogliptin alleviates cardiomyocyte oxidative stress injury in mice with DCM by enhancing AURKB‐mediated transcriptional activation of NLGN2.

In summary, alogliptin could strengthen AURKB‐induced NLGN2 transcription to alleviate DCM (Figure 7). The effect of alogliptin on oxidative stress was found for the first time in DCM. The screening and identification of AURKB and NLGN2 provide potential molecular targets for deep exploration of the possible role alogliptin might play in DCM.

## Funding

Financial support was provided by the Medical Science Research Project of Hebei (no. 20250068).

## Conflicts of Interest

The authors declare no conflicts of interest.

## Supporting information


**Figure S1:** Echocardiographic analysis of DCM mice following treatment with alogliptin (A), or combined with sh‐AURKB (B), and OE‐NLGN2 gene intervention (C).

## Data Availability

The data that support the findings of this study are available from the corresponding author upon reasonable request.
