# Supplementary material for: Alogliptin Reduces Oxidative Stress in Cardiomyocytes and Ameliorates Diabetic Cardiomyopathy via the AURKB/NLGN2 Signaling
Source: Kaohsiung J Med Sci. 2025 Dec 22;42(6):e70149. doi: 10.1002/kjm2.70149 (PMC13248775; doi:10.1002/kjm2.70149)
Supplement: Supplementary file 1 — Figure S1: Echocardiographic analysis of DCM mice following treatment with alogliptin (A), or combined with sh‐AURKB (B), and OE‐NLGN2 gene intervention (C). [file KJM2-42-e70149-s001.docx]

**Supplementary Figure 1**

**
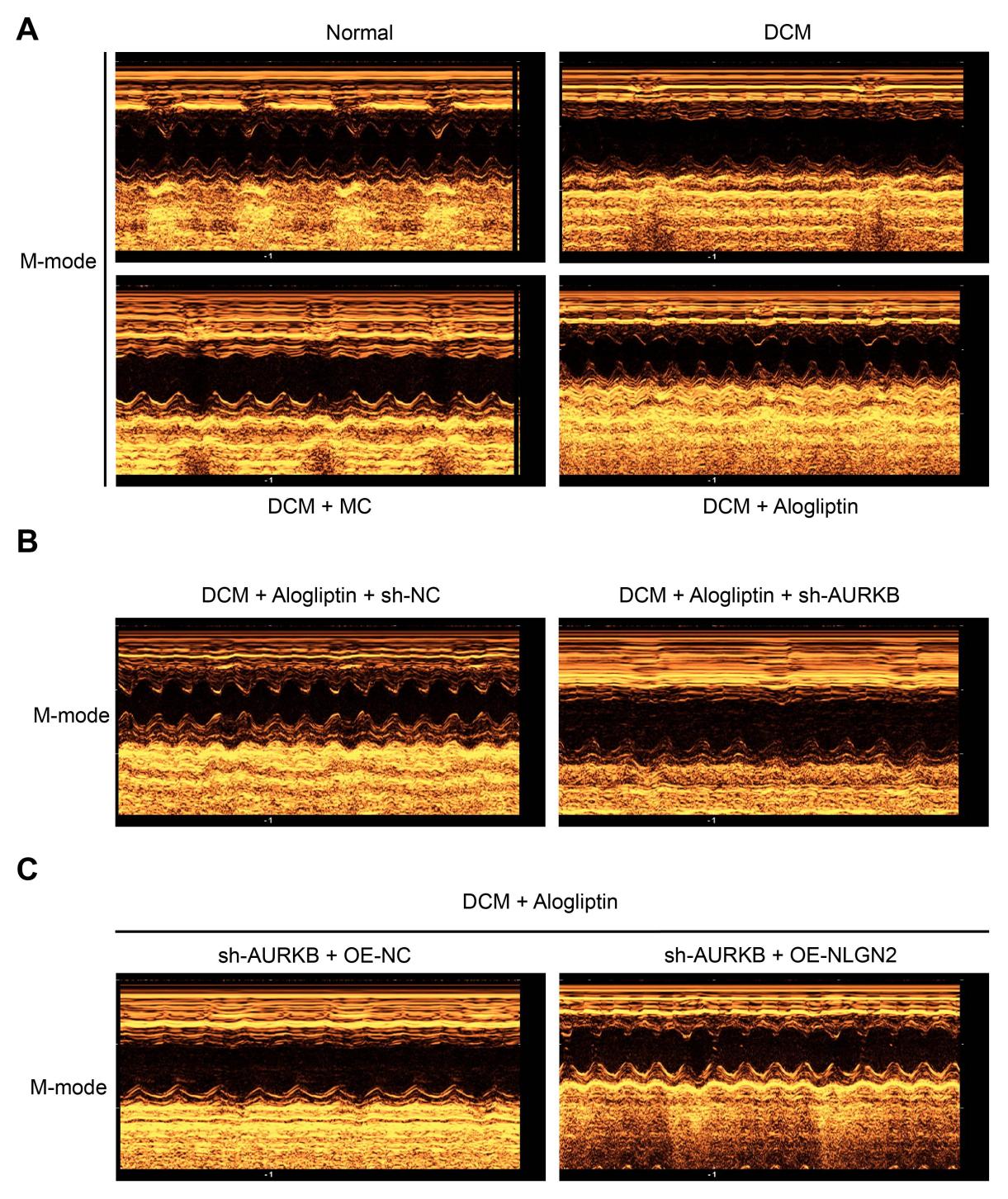
**

**Supplementary Figure 1** Echocardiographic analysis of DCM mice following treatment with alogliptin (A), or combined with sh-AURKB (B), and OE-NLGN2 gene intervention (C).
